# Dr. William D. Jenks

**Published:** 1877-05

**Authors:** 


### OBITUARY.

---

Dr. Wm. D. Jenks, a venerable and well-known citizen of Frederck City, Md., was found dead in his bed at his residence on the morning of May 1st. He practiced dentistry in Frederick for thirty years, and was very popular on account of his skill, courteous manners, &c. He was postmaster of that city during the first term of Mr. Lincoln's administration. Being greatly interested in discoveries of science, his late years have been devoted almost wholly to his library in reading journals and studying reports. He was a native of Massachusetts, was aged 85 years, a man of great benevolence, high morality and unswerving integrity, He was a member of the Masonic fraternity over sixty years.

